# Association between perioperative fluid administration and postoperative outcomes: a 20-year systematic review and a meta-analysis of randomized goal-directed trials in major visceral/noncardiac surgery

**DOI:** 10.1186/s13054-021-03464-1

**Published:** 2021-02-01

**Authors:** Antonio Messina, Chiara Robba, Lorenzo Calabrò, Daniel Zambelli, Francesca Iannuzzi, Edoardo Molinari, Silvia Scarano, Denise Battaglini, Marta Baggiani, Giacomo De Mattei, Laura Saderi, Giovanni Sotgiu, Paolo Pelosi, Maurizio Cecconi

**Affiliations:** 1 Humanitas Clinical and Research Center – IRCCS, Rozzano, MI Italy; 2 Department of Biomedical Sciences, Humanitas University, Pieve Emanuele, MI Italy; 3 Anesthesia and Intensive Care, San Martino Policlinico Hospital, IRCCS for Oncology and Neuroscience, Genoa, Italy; 4 Department of Surgical Sciences and Integrated Diagnostic (DISC), University of Genoa, Genoa, Italy; 5 Anesthesia and Intensive Care Medicine, Maggiore Della Carità University Hospital, Novara, Italy; 6 Anesthesia and Intensive Care Medicine, Azienda Sanitaria Universitaria Integrata Udine, Udine, Italy; 7 Clinical Epidemiology and Medical Statistics Unit, Department of Medical, Surgical and Experimental, University of Sassari, Sassari, Italy

**Keywords:** Fluids, Surgery, Systematic review, Metanalysis, Perioperative goal-directed therapy, Postoperative complications

## Abstract

**Background:**

Appropriate perioperative fluid management is of pivotal importance to reduce postoperative complications, which impact on early and long-term patient outcome. The so-called perioperative goal-directed therapy (GDT) approach aims at customizing perioperative fluid management on the individual patients’ hemodynamic response. Whether or not the overall amount of perioperative volume infused in the context of GDT could influence postoperative surgical outcomes is unclear.

**Methods:**

We conducted a systematic review and meta-analysis of randomized controlled trials (RCTs) comparing the efficacy of GDT approach between study population and control group in reducing postoperative complications and perioperative mortality, using MEDLINE, EMBASE and the Cochrane Controlled Clinical trials register. The enrolled studies were grouped considering the amount infused intraoperatively and during the first 24 h after the admission in the critical care unit (perioperative fluid).

**Results:**

The metanalysis included 21 RCTs enrolling 2729 patients with a median amount of perioperative fluid infusion of 4500 ml. In the studies reporting an overall amount below or above this threshold, the differences in postoperative complications were not statically significant between controls and GDT subgroup [43.4% vs. 34.2%, *p* value = 0.23 and 54.8% vs. 39.8%; *p* value = 0.09, respectively].

Overall, GDT reduced the overall rate of postoperative complications, as compared to controls [pooled risk difference (95% CI) = − 0.10 (− 0.14, − 0.07); Chi_2_ = 30.97; *p *value < 0.0001], but not to a reduction of perioperative mortality [pooled risk difference (95%CI) = − 0.016 (− 0.0334; 0.0014); *p *value = 0.07]. Considering the rate of organ-related postoperative events, GDT did not reduce neither renal (*p *value = 0.52) nor cardiovascular (*p *value = 0.86) or pulmonary (*p *value = 0.14) or neurological (*p *value = 0.44) or infective (*p *value = 0.12) complications.

**Conclusions:**

Irrespectively to the amount of perioperative fluid administered, GDT strategy reduces postoperative complications, but not perioperative mortality.

**Trial Registration:**

CRD42020168866; Registration: February 2020

https://www.crd.york.ac.uk/prospero/display_record.php?RecordID=168866

## Introduction

Postoperative complications occur in a significant proportion of patients undergoing surgery [1–3], leading to mortality of about 4% in Europe [4], and having a significant impact on long-term morbidity and, in turn, on health and financial systems [5, 6]. Several aspects including preoperative frailty, intraoperative management and events and postoperative care may influence the risk of developing postoperative complications.

In this context, the optimization of perioperative fluid management plays a key role in maintaining tissue fluid and electrolyte homeostasis and euvolemia, while avoiding inadequate tissue perfusion and fluid overload, which have been both associated with worse clinical outcomes in surgical patients [7–13]. The most effective perioperative fluid management is unclear [14–17]. The Enhanced Recovery After Surgery (ERAS) pathways to support early recovery among patients undergoing major surgery recommend a restrictive approach aiming for the perioperative “zero-balance” [14]. In contrast, recent findings suggest that this approach could be detrimental, suggesting a moderately positive fluid balance of 1 to 2 L at the end of surgery [16].

However, regardless of the definitions adopted, perioperative fluid balance may result from either preoperative fixed fluid targets (i.e., overall fluid balance below a predetermined cutoff), or, as part of a protocol-based fluid administration. The so-called perioperative goal-directed therapy (GDT) is based on the purpose of balancing the increased oxygen demand during surgery, by the use of flow-based hemodynamic parameters, to achieve specific hemodynamic endpoints rather than a predetermined perioperative fluid balance [8, 18]. Adopting a GDT approach, perioperative fluid balance is the effect of the individual response to fluid administration, being titrated on the hemodynamic response to each fluid bolus. However, the evidence regarding the effectiveness of perioperative GDT approaches is still inconclusive [8, 19–21].

We conducted a systematic review and meta-analysis of RCTs to assess whether the amount of perioperative volume administered by means of a GDT approach (defined as the quantity infused in intraoperative time and the first 24 h after the admission to the critical care unit) could influence postoperative outcomes. To address this point, we stratified the included studies considering the median amount of perioperative fluid given to patients receiving GDT, as compared to the controls. Secondarily, we assessed the overall effect of GDT on postoperative overall and organ-specific complications (*i.e.* renal, cardiovascular, pulmonary, neurological and infective), and perioperative mortality.

## Material and method

We adhered to the *Preferred Reporting Items for Systematic Reviews and Meta-Analysis-Protocols* (PRISMA-P) guidelines [22] (Additional file 1: Table S1). The study protocol was registered with the *International Prospective Register of Systematic Reviews* (PROSPERO) in June 2020 (CRD42020168866).

### Data sources and search strategy

A systematic literature search was performed including the following databases: PUBMED®, EMBASE® and the Cochrane Controlled Clinical trials register. The search was performed using the terms: ('goal-directed therapy' OR ('goal directed' AND ('therapy'/exp OR therapy)) OR 'goal-oriented therapy' OR ('goal oriented' AND ('therapy'/exp OR therapy)) OR 'goal-targeted therapy' OR ('goal targeted' AND ('therapy'/exp OR therapy))) AND ('surgery'/exp OR surgery) with filters for randomized trials.

Articles written in English and published from January 1, 2000, to December 31, 2019, in indexed scientific journals were considered. Editorials, commentaries, letters to editor, opinion articles, reviews, meeting abstracts were excluded. Only RCTs recruiting adult surgical populations using GDT approaches, reporting on morbidity (rate of postoperative complications) and/or mortality as primary or secondary outcomes were included. Studies focused on cardiac, trauma/orthopedic, pediatric, obstetric or neurosurgeries were excluded. References of selected papers, review articles, commentaries and editorials on this topic were also reviewed to identify other studies of interest missed during the primary search. When multiple publications of the same research group/center described potentially overlapping cohorts, the more recent publications were selected.

GDT strategy was defined as a modality of perioperative treatment including the use of both (1) hemodynamic monitoring and (2) therapies (fluids and/or inotropes and vasopressors, alone or together) aimed at manipulating hemodynamic parameters during the perioperative period to achieve a predetermined hemodynamic endpoint(s). The step-by-step perioperative protocol based on patient-specific hemodynamic data retrieved from both a hemodynamic monitor (irrespective to the invasiveness) or surrogates of peripheral delivery/extraction of oxygen (*i.e*. lactate, central venous oxygen saturation, capillary refill time) had to be detailed in the selected studies.

The GDT goals might include both the optimization of either systemic flow or pressure parameters (*i.e.*, mean systemic pressure or stroke volume), or hemodynamic indexes (i.e*.*, stroke or pulse pressure variations). Only RCTs comparing GDT strategy versus a single control population were selected. We considered perioperative fluid administration as the overall amount of fluids infused in a period including intraoperative time and the first 24 h after admission to a critical care unit [15].

### Data abstraction and quality assessment

Three couples of examiners independently evaluated titles and abstracts. The articles were then subdivided into three subgroups: “included” and “excluded” (if the two examiners agreed with the selection) or “uncertain” (in case of disagreement). In the case of “uncertain” classification, discrepancies were resolved by further examination performed by two expert authors (A.M. and C.R.). We used a standardized electronic spreadsheet (Microsoft Excel, V 14.4.1; Microsoft, Redmond, WA) to extract data from all included studies, recording: trial characteristics (*i.e.* number of centers, country), patient population (*i.e.* demographics, type of surgery, baseline illness severity scores), intraoperative monitoring and interventions (*i.e.* mechanical ventilation characteristics, monitoring technology used, goal-directed therapy targets, type and amount of fluid) and clinical outcomes (*i.e.* mortality, morbidity related to organ-specific function or infections) (Additional file 1: Table S2). When necessary, the corresponding authors of the included studies were contacted to obtain missing data related to trial demographics, methods and outcomes.

### Risk of bias assessment in the included studies.

The internal validity of the included studies was assessed by two expert authors (A.M. and C.R.), and discrepancies were resolved by a third author (M.C.) by using the RoB 2: a revised Cochrane Collaboration’s risk-of-bias tool for randomized trials [23]. The RoB 2 considers five bias domains: (1) the randomization process; (2) the deviations from intended interventions; (3) missing outcome data; (4) measurement of the outcome; (5) selection of the reported results. Finally, overall risk of bias was calculated and, accordingly, studies were classified as high-risk/some concerns/low-risk.

### Statistical analysis

Descriptive analysis was carried out: The statistical unit of observation for all the selected variables was the single study and not the patient. Means with standard deviations (SD) described for continuous variables.

The meta-analysis included only those studies reporting the rate of overall perioperative complications, according to the definition adopted in each study (i.e., overall, severe, not-severe). For the primary outcome, the association between perioperative fluid administration and complications was assessed considering the median fluid dose given to the subgroups of patients receiving GDT, as compared to controls, and predefined cutoffs of postoperative complications frequencies (i.e., < 30%; 30–50%; > 50%). For the secondary outcomes, we considered the effect of GDT approach on (1) postoperative overall rate of complications (number of patients having at least one complication); (2) organ-related events [renal, cardiac, pulmonary non-infective (i.e., pulmonary embolism), neurological and infective (including either sepsis/septic shock or organ-specific infections)]; (3) perioperative mortality.

Publication bias was graphically evaluated using funnel plots. Heterogeneity was measured using *Q* and *I*^2^ tests, which were considered significant when the *p *value was < 0.1 and *I*^2^ > 50%. Random or fixed effect models were used based on the expected heterogeneity. According to Higgins et al. [24], *I*^2^ values around 25%, 50% and 75% represented low, moderate and high heterogeneity.

The statistical software STATA® version 16 (StataCorp, College Station, TX, USA) and StatsDirect version 3.2.7 were used to perform all the statistical computations.

## Results

**Fig. 1:**
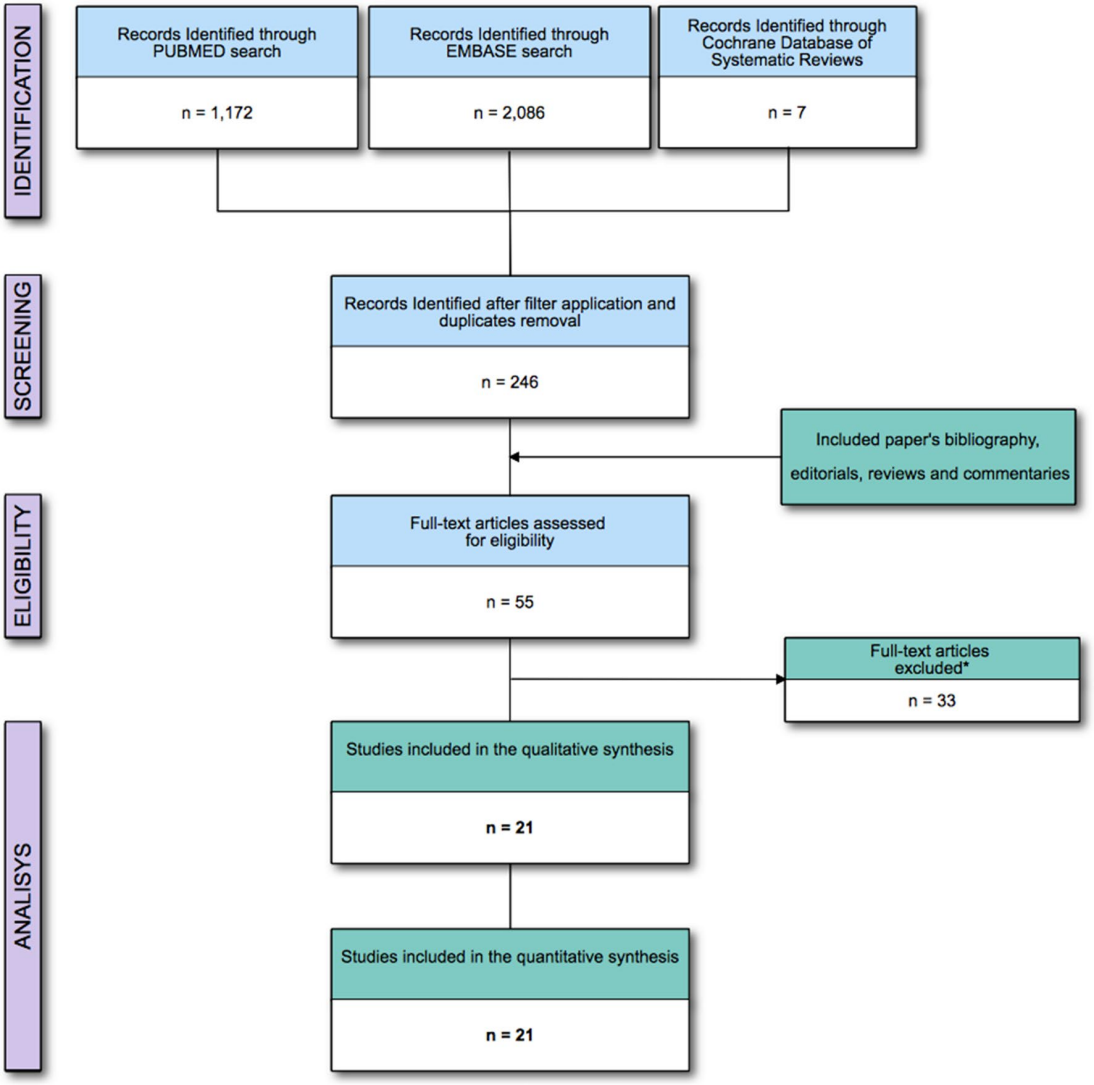
Flow of the studies. * = Not fitting eligibility criteria full-text articles excluded a reported in the Additional file 1: Table S3

The electronic search identified 1,834 titles after removing duplicate studies. Experts evaluated and solved the inclusion of 25 (1.3%) potentially relevant studies because of disagreement between the examiners. The meta-analysis was performed on 21 RCTs enrolling 4753 and analyzing 2729 patients (Fig. 1 and Table 1). Excluded studies are reported in Additional file 1: Table S3. The bias risk assessment reported “low risk” for 5 (22.7%) and “some concerns” for 16 (76.2%) of the included studies, mostly related to the selection of the reported results (Additional file 1: Figure S1).

**Table 1:** Summary of the included GDT studies

First author	Year	Surgery	Pt. Enrolled *n*	Pt. Analyzed *n* (%)	GDT *n* (%)	Controls *n* (%)	ASA I *n* (%)		ASA II *n* (%)		ASA III-IV *n* (%)	
							GDT	Controls	GDT	Controls	GDT	Controls
Conway [41]	2002	Abdominal surgery	57	57 (100)	29 (50.9)	28 (49.1)	NA	NA	NA	NA	NA	NA
Wakeling [42]	2005	Abdominal surgery	178	128 (71.9)	64 (50)	64 (50)	NA	NA	NA	NA	NA	NA
Pearse [30]	2005	Mixed	160	122 (76.3)	62 (50.8)	60 (49.2)	NA	NA	NA	NA	NA	60 (100)
Lobo [26]	2006	Mixed	72	50 (69.4)	25 (50)	25 (50)	NA	NA	NA	NA	NA	NA
Donati [31]	2007	Mixed	324	135 (41.7)	68 (50.4)	67 (49.6)	0 (0.0)	0 (0.0)	9 (13.3)	11 (16.4)	59 (86.8)	56 (83.6)
Benes [25]	2010	Mixed	215	105 (48.8)	51 (48.6)	54 (51.4)	0 (0.0)	0 (0.0)	14 (23.3)	11 (18.3)	46 (76.4)	49 (81.7)
Mayer [43]	2010	Abdominal surgery	102	60 (58.8)	30 (50)	30 (50)	NA	NA	NA	NA	30 (100)	30 (100)
Brandstrup [32]	2012	Abdominal surgery	185	150 (81.1)	71 (47.3)	79 (52.7)	26 (37)	20 (25)	37 (52)	43 (54)	8 (11)	16 (20)
Salzwedel [27]	2013	Mixed	180	160 (88.9)	79 (49.4)	81 (50.6)	NA	NA	NA	NA	33 (41.3)	33 (40.7)
McKenny [44]	2013	Gynecological surgery	113	101 (89.4)	51 (50.5)	50 (49.5)	NA	NA	NA	NA	NA	NA
Scheeren [28]	2013	Mixed	64	52 (81.3)	26 (50)	26 (50)	0 (0.0)	0 (0.0)	0 (0.0)	0 (0.0)	26 (100)	26 (100)
Srinivasa [45]	2013	Abdominal surgery	98	74 (75.5)	37 (50)	37 (50)	5 (13)	5 (13.5)	20 (54)	15 (40.5)	12 (32)	17 (46)
Pearse [19]	2014	Mixed	1735	734 (42.3)	368 (50.1)	366 (49.9)	21 (5.7)	24 (6.6)	201 (54.5)	176 (48.1)	146 (39.8)	166 (45.3)
Phan [46]	2014	Abdominal surgery	131	100 (76.3)	50 (50)	50 (50)	NA	NA	NA	NA	NA	NA
Ackland [47]	2015	Mixed	204	187 (91.7)	95 (50.8)	92 (49.2)	NA	NA	NA	NA	NA	NA
Correa-Gallego [48]	2015	Abdominal surgery	468	135 (28.8)	69 (51.1)	66 (48.9)	NA	NA	NA	NA	NA	NA
Weinberg [34]	2017	Abdominal surgery	68	52 (76.5)	26 (50)	26 (50)	NA	NA	NA	NA	NA	NA
Gomez-Izquierdo [49]	2017	Abdominal surgery	196	128 (65.3)	64 (50)	64 (50)	6 (9)	8 (12)	42 (66)	38 (60)	16 (25)	18 (28)
Wu [50]	2017	Neurological surgery	111	63 (56.8)	33 (52.4)	30 (47.6)	13 (40)	9 (30)	20 (60)	21 (70)	0 (0.0)	0 (0.0)
Zhao [33]	2018	Abdominal surgery	88	88 (100)	44 (50)	44 (50)	NA	NA	31 (70)	29 (66.6)	13 (30)	15 (33.1)
Weinberg [29]	2019	Abdominal surgery	61	48 (78.7)	24 (50)	24 (50)	NA	NA	NA	NA	18 (75)	21 (88)

Pt., patients; *N*; number of patients; NA, data not available; ASA, American Society of Anesthesiologists physical status; GDT, goal-directed therapy

### General characteristics of the GDT subgroups and perioperative fluid administration.

Patient characteristics were consistent among the studies. Patients receiving GDT had a mean (SD) age of 62 (5) years (males = 59%), a mean (SD) body mass index of 26 ± 2 kg/m^2^, and had a mean (SD) surgical time of 238 (91) minutes. In the controls subgroup, mean (SD) age was 66 (6) years (males = 56.4%) with mean (SD) body mass index of 26 ± 2 and had a mean (SD) surgical time of 236 (81) minutes (see also Tables 1 and 2).

**Table 2:** Characteristics of GDT/control subgroups hemodynamic protocol

	Group	Bolus (amount)	Bolus (type)	Vasopressor (type, % of pt.)	Hemodynamic goal	Transfusion (% of pt.)	Hemodynamic monitor
Conway [41]	GDT	3 ml/kg	Voluven®	NA	SV > 10% after FC FTC < 0.35 s	NA	ODP (TECO2®)
	Control	NA	NA	NA	NA	NA	Standard monitoring
Wakeling [42]	GDT	250 ml	Haemaccel® Gelofusine ®	NA	Repeated FC until SV response > 10% or CVP > 3 mmHg	NA	ODP—CardioQ
	Control	Standard care	Standard care	NA	CVP 12- 15 mmHg Oxygen Delivery Index > 600 ml/min/m^2^	NA	ODP—CardioQ
Pearse [30]	GDT	250 ml	Gelofusine ®	Dopexamine (all)	SV response > 10% for 20 min CVP increase > 2 mmHg for 20 min	NA	LiDCOplus
	Control	250 ml	Gelofusine ®	NA	CVP increase of at least 2 mmHg for 20 min	NA	Standard monitoring
Lobo [26]	GDT	1000 ml 500 ml	Crystalloids Gelatine	Dobutamina; 8%	Oxygen Delivery Index < 600 ml/min/m^2^ PAOP < 16 mmHg	44%	PAC
	Control	1000 ml 500 ml	Crystalloids Gelatine	Dobutamina; 100%	Oxygen Delivery Index < 600 ml/min/m^2^ PAOP < 16 mmHg	48%	PAC
Donati [31]	GDT	NA	Colloids UN (250–1000 ml)	Dobutamine; 44.1%	O_2_ER < 27% MAP > 80 mm Hg UO > 0.5 ml/kg/h CVP 8–12 cmH_2_0	NA	O_2_ER
	Control	NA	Colloids UN (250–1000 ml)	Dobutamine; 4.5%	MAP > 80 mm Hg UO > 0.5 ml/kg/h CVP 8–12 cmH_2_0	NA	Standard monitoring
Benes [25]	GDT	3 ml/kg	Voluven ® Tetraspan ®	Norepinephrine; 13.7% Dobutamine; 1.9%	SVV < 10% CI/CVP changes after FC	NA	Vigileo/FT
	Control	NA	Colloids UN Crystalloids UN	Norepinephrine, 11.1% Dobutamine, 0%	MAP > 65 mm Hg UO > 0.5 ml/kg/h CVP 8–115 cmH_2_0	NA	Standard monitoring
Mayer [43]	GDT	500 ml (first) 250 ml (further)	Cristalloyds UN Colloids UN	Norepinephrine (%NA) Dobutamine (%NA)	CI < 2.5 L/min/m^2^ MAP > 65 mm Hg SVI > / < 35 ml/min/m^2^	NA	Vigileo/FT
	Control	500 ml (first) 250 ml (further)	Cristalloyds UN Colloids UN	NA	MAP 65–90 mm Hg UO > 0.5 ml/kg/h CVP 8–12 cmH_2_0	NA	Standard monitoring
Brandstrup [32]	GDT	200 ml	Voluven ®	Different vasopressors (%NA)	Repeated FC until SV response > 10%	NA	ODP—CardioQ
	Control	200 ml	Voluven ®	Different vasopressors (%NA)	MAP > 60 mmHg Hematocrit 25 and 35%	NA	ODP—CardioQ
Salzwedel [27]	GDT	NA	NA	Norepinephrine, 32.9% Phenylephrine,0% Ephedrine, 13.9% Dobutamine, 41.8%	PPV < 10% CI > 2.5 L/min/m^2^ MAP > 65 mmHg	NA	ProAQT
	Control	NA	NA	Norepinephrine, 3.5% Phenylephrine, 4.9% Ephedrine, 9.8% Dobutamine, 0%	AD	NA	Standard monitoring
McKenny [44]	GDT	3 ml/kg	Voluven ®	NA	Repeated FC until SV response > 10%	8; 16%	ODP
	Control	Standard care	AD	NA	AD	8; 16%	ODP
Scheeren [28]	GDT	200 ml	Voluven ®	Norepinephrine (% NA)	Repeated FC if SVV > 10% Repeated FC until SV response > 10%	NA	Vigileo/FT
	Control	Standard care	Colloids UN Crystalloids UN	Norepinephrine (% NA)	Standard care	NA	Vigileo/FT
Srinivasa [45]	GDT	7 ml/kg FC first 3 ml/kg other	Gelofusine®	83.7% (type UN)	FTC 0.35 – 0.4 s SV after FC > 10%	13, 35%	ODP—CardioQ
	Control	Up to 1500 ml PlasmaLyte® Up to 1500 ml Gelofusine®		91.8% (type UN)	Standard care	12, 32%	ODP—CardioQ
Pearse [19]	GDT	250 ml	Colloids UN	Bolus 82.2% Infusion 28.1%	MAP 60–100 mmHg SV optimization	NA	LiDCOrapid
	Control	Standard care	Standard care	Bolus 74.8% Infusion 30%	Standard care	NA	NA
Phan [46]	GDT	250 ml	Voluven® Gelofusine® 4% human albumin	NA	Hypotension SVI < 35 ml/m^2^ FTc < 0.3 s	None	ODP
	Control	Blood loss replacement Hypotension not responsive to vasopressor	Ringer lactate UN Colloids	NA	none	None	Standard monitoring
Ackland [47]	GDT	NA	Gelatine	Vasopressor 18 (19%)	Oxygen Delivery Index pre-postoperative Repeated FC until SV response > 10%	25 (26%)	LiDCOplus
	Control	NA	Gelatine	Vasopressor 21 (23%)	ScVO_2_ > 65% MAP > 60 mm Hg UO > 0.5 mL/kg/h Oxygen Delivery Index	16 (17%)	LiDCOplus
Correa-Gallego C	GDT	1:1 blood loss replacement Albumin infusion		(type NA) 58%	SVV to a value ≤ 2 SD from baseline	6%	EV1000
	Control	1:1 blood loss replacement 6 ml/kg/hr Crystalloids		(type NA) 58%	Hypotension systolic BP < 90 mmHg, Urine output < 25 ml/h	2%	EV1000
Weinberg	GDT	250 mL	Hartmann or PlasmaLyte 4% or 20% albumin	NA	SVV < 20% MAP < 20% of baseline CI > 2 L/kg/m^2^	NA	EV1000
	Control	AD	Hartmann or PlasmaLyte 4% or 20% albumin	NA	AD	NA	EV1000
Gomez-Izquierdo [49]	GDT	200 mL	Voluven®	NA	SV optimization	NA	ODP
	Control	AD	Ringer Lactate Voluven®	NA	AD	NA	ODP
Wu [50]	GDT	50 ml	Colloids (UN)	Ephedrine, phenylephrine; dobutamine	SVV < 12%	NA	Vigileo/FT
	Control	50 ml	Colloids (UN)	Ephedrine, phenylephrine	CVP > 8 mmHg MAP < 80% of baseline value	NA	Vigileo/FT
Zhao [33]	GDT	200 mL	Different Colloids	MAP/CI guided	SVV < 15% MAP ≥ 65 mmHg CI > 2.5 L/min/m^2^	NA	Vigileo/FT
	Control	AD	AD	NA	NA	NA	Standard monitoring
Weinberg [29]	GDT	250 mL	AD with Crystalloids or Colloids	MAP/CI guided	SVV < 20%/15% MAP of 20% basal CI > 2.2 L/min/m^2^	NA	EV1000
	Control	AD	AD with Crystalloids or Colloids	Discretion of the anesthetist	CVP > 8 mmHg during dissection and hepatic transection stages	NA	EV1000

NA, data not available; UN, unspecified; AD; at the discretion of the care-giving anesthesiologist; GDT, patients’ subgroup receiving goal-directed therapy; mL, milliliters; SV, stroke volume; FTC, corrected flow time; ODP, esophgeal doppler probe; O_2_ER, oxygen extraction; MAP, mean arterial pressure; UO, urine output; CVP, central venous pressure; SVV, stroke volume variation; FC, fluid challenge; CI; cardiac index; PAC, pulmonary artery catheter; PAOP; pulmonary artery occlusion pressure

Vigileo/FT; Vigileo/Flow Track: Edwards Lifesciences, Irvine, CA, USA

CardioQ: DP12 probe; Pharmaco NZ, Auckland, New Zealand

ProAQT: PULSION Medical Systems SE, Munich, Germany

LiDCOrapid/ LiDCOplus: LiDCO, Cambridge, UK

The summary of the intraoperative amount of fluids infused is reported in Table 2. Intraoperatively, the GDT subgroup received a mean (SD) of 1632 (928) ml of crystalloids and of 1053 (603) ml of colloids (51% of the overall amount). In contrast, controls received a mean (SD) of 1977 (1142) ml of crystalloids, and of 758 (586) ml of colloids (44% of the overall amount). Nine studies [25–33] reported a mean (SD) intraoperative blood transfusion of 320 ml (297) and 340 ml (299) in GDT and controls, respectively. Only three studies [27, 29, 34] reported the cumulative fluid balance of GDT and control groups during the first two postoperative days (Additional file 1: Table S4).

### Effect of fluid administration on the rate of complications

The median amount of perioperative fluid infusion in the included studies was 4500 ml. Differences in the incidence (95% CI) of postoperative complications between patients receiving GDT and controls in those studies reporting a volume below or above this thresholds were not significant [43.4% (34.2; 54.9) vs. 34.2% (22.5; 45.8), *p *value = 0.23 and 54.8% (42.5; 67.0) vs. 39.8% (26.5; 53.1); *p *value = 0.09, respectively].

In the subgroup of those studies reporting an overall rate of complication < 30%, the total amount of perioperative fluids was comparable between patients receiving GDT and controls s [3474 (2313; 4635) vs. 4236 (2765; 5708); *p *value = 0.40]. The same result was observed for the subgroups of those studies reporting an overall rate of complication between 30 and 50% [4534 (2905; 6162) vs. 4153 (3053; 5254); *p *value = 0.64] or above 50% [4723 (3714; 5732) vs. 5425 (3886; 6966); *p *value = 0.36].

### Postoperative outcomes: complications and perioperative mortality

**Fig. 2:**
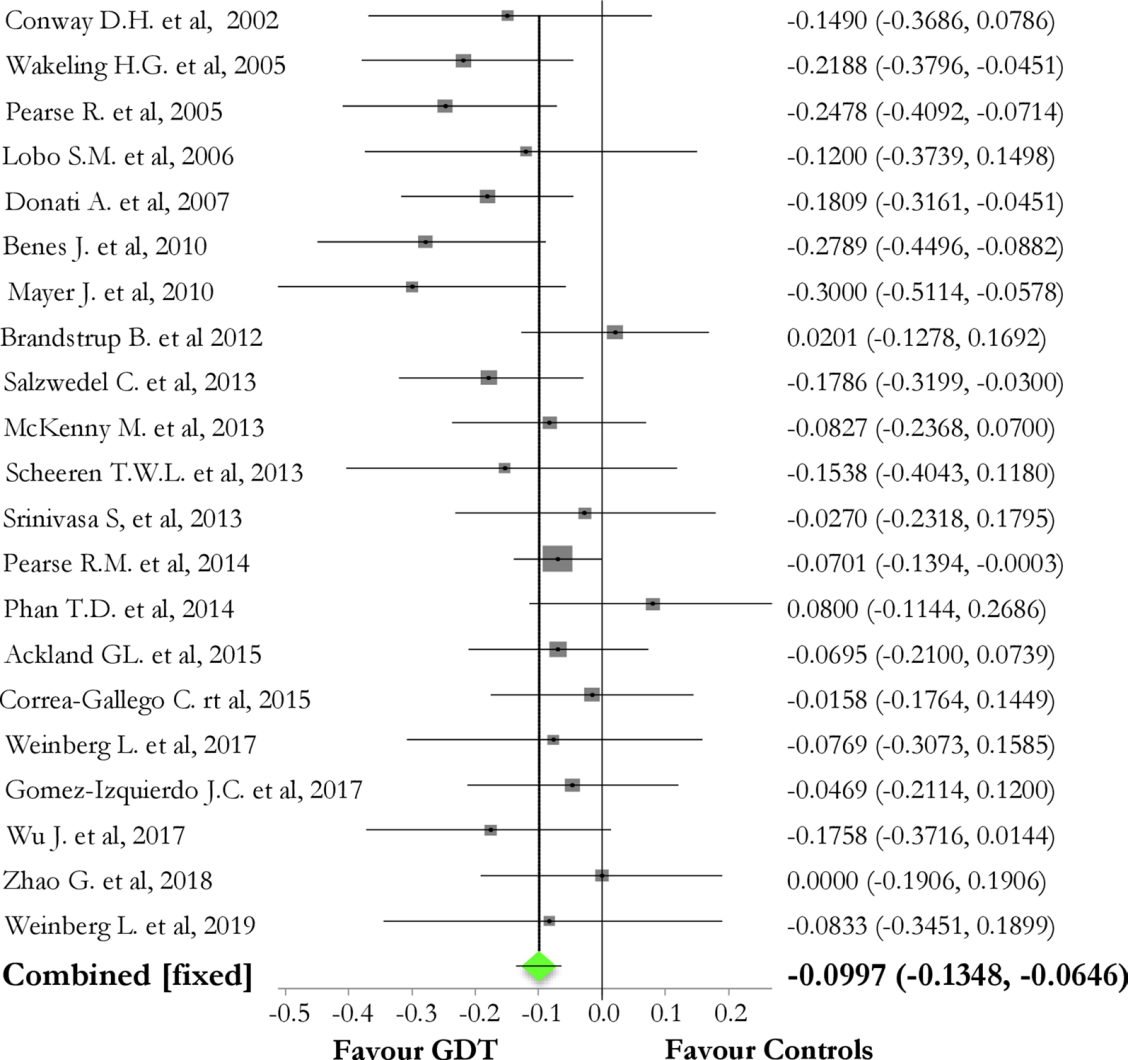
Forest plot of the effect of goal-directed therapy (GDT) in protocol group versus controls on the rate of postoperative complications. The pooled risk difference is in favor of GDT group [pooled risk difference GDT vs. controls (95% CI) = − 0.10 (− 0.14, − 0.07); Chi_2_ = 30.97, *p *value < 0.00001; *I*_2_ (95% CI) = 19% (0–52.1%); bias assessment funnel plot is reported in Additional file 1: Figure S2]

GDT reduced the rate of postoperative complications (Fig. 2 and Additional file 1: Figure S2) [pooled risk difference GDT vs. controls (95% CI) = − 0.10 (− 0.14, − 0.07); Chi_2_ = 30.97, *p *value < 0.00001; *I*_2_ (95% CI) = 19% (0–52.1%)]. Considering the rate of organ-related postoperative events, GDT did not reduce neither renal (*p *value = 0.52), nor cardiovascular (*p *value = 0.86) or pulmonary (*p *value = 0.14) or neurological (*p *value = 0.44) or infective (*p *value = 0.12) complications (Additional file 1: Tables S5–S9).

**Fig. 3:**
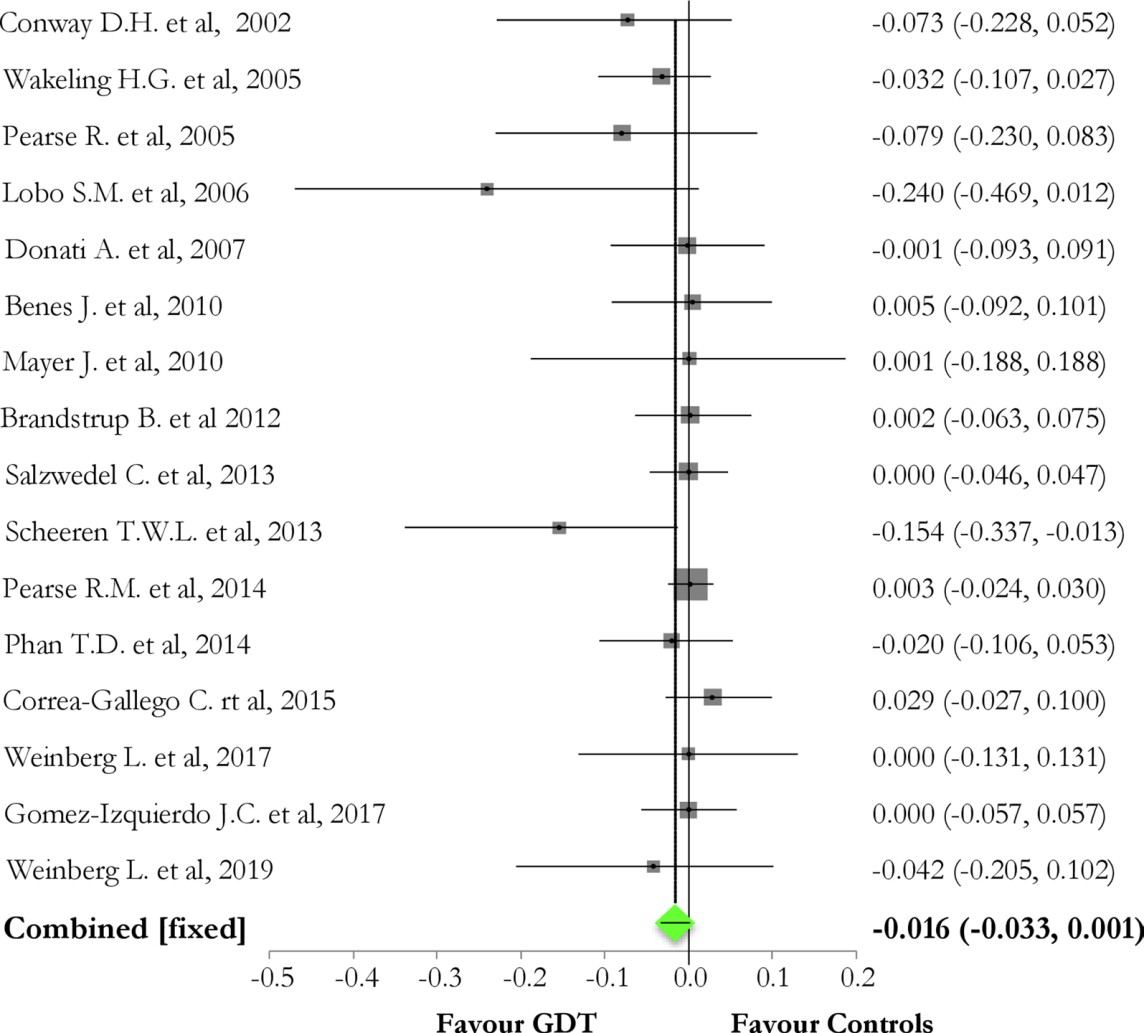
Forest plot of the effect of goal-directed therapy (GDT) in protocol group versus controls on perioperative mortality. The pooled risk difference between GDT and control groups was not statistically significant [pooled risk difference GDT vs. controls (95% CI) = − 0.016 (− 0.0334; 0.0014); Chi_2_ = 3.23, *p *value = 0.07; *I*_2_ = 18% (0–54.8%); bias assessment funnel plot is reported in Additional file 1: Figure S3]

In the 16 studies reporting perioperative mortality, GDT strategy did not show any benefits (Fig. 3 and Additional file 1: Figure S3) [pooled risk difference GDT vs. controls (95%CI) = − 0.016 (− 0.0334; 0.0014); Chi_2_ = 3.23, *p *value = 0.07; *I*_2_ 18% (0–54.8%)].

## Discussion

The main findings of this systematic review and meta-analysis can be summarized as follows: (1) GDT significantly reduces postoperative complications but not mortality; (2) the effect of GDT on postoperative complications was not modified by the overall amount of fluid infused in the perioperative period.

To the best of our knowledge, this is the most updated and complete systematic review including RCTs comparing patients managed with GDT vs. controls in the perioperative period, as compared to other meta-analyses [8, 9, 19].

Postoperative complications are common after major surgery and represent an important financial and social burden [5, 6]. The optimization of fluid management has been extensively studied as a potential adjustable perioperative factor, by adopting specific protocols aimed at optimizing predetermined hemodynamic endpoints [7–13]. This step-by-step GDT process should, in principle, prevent fluid overload (irrespective of the overall amount administered), by closely monitoring the effects on predefined flow or pressure variables of each bolus administered, as long as the individual plateau is achieved [35, 36]. In other words, during GDT, perioperative overall fluid administration is one of the tools to achieve a predefined endpoint and not the endpoint itself, and its titration should reduce postoperative complications, irrespective of the dose achieved.

In the field of perioperative fluid administration, summarizing the evidence available in the literature into clear clinical suggestions for the daily clinical practice is rather complex. On the one hand, there is a tendency towards a more restrictive approach (as supported by the ERAS pathways [14]). On the other, a recent large RCT performed by Myles et al. challenged this concept, and showed that a median intravenous-fluid intake of 3.7 l as compared to 6.1 l did not affect the rate of disability-free survival at one year, being also associated with higher rates of acute kidney injury, surgical-site infection and renal-replacement therapy [15]. The negative results of this trial could be partially explained by the lack of the individualization of fluid administration, which may have contributed to the renal damage, together with the use of vasopressors, low blood pressure and, possibly, dehydration.

Interestingly, GDT trials performed in the period 2000–2010 ago showed important improvements in postoperative morbidity, as compared to more recent ones (Fig. 2). Factors influencing this discrepancy may be related to a different approach to the perioperative fluid balance, along with the advances in all the ERAS components (i.e., avoidance of opioids, early feeding and ambulation and others) and in surgical care (i.e*.* more minimally invasive approaches). Our results add to this discussion by suggesting that, in those perioperative scenarios expected to be managed with large amounts of fluids, the use of a GDT approach should be encouraged, since a trend towards the reduction of postoperative complications was shown.

As a matter of fact, by including studies reporting an overall median rate of postoperative complications > 30%, GDT approach reduces overall postoperative complications [pooled risk difference (95% CI) = − 0.10 (− 0.14, − 0.07); *p *value < 0.00001], confirming data retrieved from previous findings meta-analyses [8, 9, 19], and encouraging the use of the GDT approach for managing high risk surgical patients.

In this setting, the definition of postoperative complications is crucial, but it is far from being standardized. We only included trials performed in a relatively short timeline, avoiding outdated trials, which may not be representative of current clinical practice.

However, the results of the GDT on overall or organ-related rate of postoperative complications could be related to the quality of data reported in the studies. In fact, both the analyses of the rate of overall postoperative complications and mortality considered the rate of patients who had at least one complication or who died. In contrast, the analysis of organ-related complications considered the number of specific organ-related events. This latter evaluation.

is potentially biased by (1) the definition adopted in the period of enrolment (i.e., the definition of postoperative renal or cardiac dysfunction have been continuously updated [37, 38]); (2) the overlapping of some clinical definitions (i.e., the occurrence of postoperative acute respiratory distress syndrome may be considered as both pulmonary or infective complication); (3) data could be biased by those patients having more than one complication; (4) the gravity of the events potentially biasing the comparability and consistency of pooled data (i.e., sepsis or would infection are both postoperative infective events).

On the contrary, we found no difference in perioperative mortality, despite a trend towards its reduction in the GDT subgroup (*p* = 0.07). In a previous systematic review, the use of GDT showed an overall benefit in mortality (OR 0.52, 95% CI 0.36–0.74; *p* = 0.003); however, when analyzing the population according to the surgical risk, the authors found that mortality benefit was observed only in the high risk patients subgroup (OR 0.20, 95% CI 0.09–0.41; *p* < 0.0001), whereas it had no effect on intermediate-risk patients (OR 0.83, 95% CI 0.41–1.69; *p* = 0.62) [8]. This review also included studies performed in a relatively longer timeframe, with some studies reporting high mortality rates. This could be related to the increasing improvement of overall perioperative management strategies, including fluid administration policies [35, 36].

### Strengths and Limitations of this meta-analysis.

Our study has some limitations. First, the consistency of data reporting postoperative fluid administration is also somewhat lacking. We considered the amount of fluid administration, including both colloids and crystalloids, since data regarding blood products are not reported in most of the included studies. Moreover, more comprehensive indications regarding the perioperative fluid policy adopted would result from the analysis of fluid balance, which is, unfortunately, reported in only three studies [27, 29, 34]. The effect of the type of fluids adopted in the studies may also play a role. Intraoperatively, the GDT subgroup received roughly the same amount of crystalloid and colloids, whereas controls received slightly more crystalloids (56%). Boluses of colloids have been frequently used to optimize predefined hemodynamic targets, whereas crystalloids have often been infused often as maintenance (Table 3). However, a minority of the included studies reported a significant difference in the amount of crystalloids/colloids used between GDT and control subgroups (Table 3). Despite a recent RCT comparing the use of low-molecular-weight hydroxyethylstarch vs. 0.9% saline in high-risk surgical patients showed no significant difference in postoperative outcomes [39], the debate regarding the optimal perioperative fluid is still ongoing, and the inconsistency of data did not allow any specific subgroup analysis on this topic.

**Table 3:** Summary of perioperative fluids administered in the included studies

Study	Total intraoperative fluid administration						Total perioperative Fluid administration (95% CI)	
	Colloids (ml) (95% CI/SD)		Crystalloids (ml) (95% CI/SD)		Intraoperative blood (ml)			
	GDT	Controls	GDT	Controls	GDT	Controls	GDT	Controls
Conway [41]	1960 (NA)	1325 (NA)	NA	NA	NA	NA	4522 (NA)	3770 (NA)
Wakeling [42]	2000 (NA)*	1500 (NA)	3000 (NA)	3000 (NA)	NA	NA	5000 (NA)	4500 (NA)
Pearse [30]	1907 (NA)*	930 (NA)	1204 (NA)	960 (NA)	125 (0–734)	0 (0–485)	2837 (NA)	2164
Lobo [26]	NA	NA	NA	NA	713 (458)	609 (244)	7447 (NA)	6877 (NA)
Donati [31]	1940 (673)	1805 (611)	NA	NA	260 (130)	271 (173)	4131 (NA)	4014 (NA)
Benes [25]	1425 (1000–1500)	1000 (540–1250)	2321 (1640–3002)	2459 (1529–3389)	0 (0–500)	270 (0–578)	5333 (NA)	4987 (NA)
Mayer [43]	1188 (638–1738)*	817 (350–1284)	2489 (1684–3294)	3153 (1889–4417)*	NA	NA	4528 (2211–6845)	4494 (2933–6055)
Brandstrup [32]	810 (NA)*	475 (NA)	483 (NA)	443 (NA)	78 (278)	77 (407)	6144 (NA)	5909 (NA)
Salzwedel [27]	773 (109–1437)	724 (4–1444)	2862 (1646–4078)	2680 (1526–3834)	145 (371)	224 (1036)	7053 (NA)	7597(NA)
McKenny [44]	1000 (1000–1500)*	500 (0–1000)	1000 (787–1750)	2000 (1725–2500)*	NA	NA	2620 (NA)	2881 (NA)
Scheeren [28]	1589 (1150–2028)	927 (631–1223)	NA	NA	319 (495)	685 (832)	4477 (3740–5214)	4528 (3693–5363)
Srinivasa [45]	591 (471)	297 (275)	NA	NA	NA	NA	5744 (NA)	4014 (NA)
Pearse [19]	1250 (1000–2000)	500 (0–1000)	1000 (459–2000)	2000 (1283–3000)	NA	NA	3256 (NA)	3100 (NA)
Phan [46]	500 (250–750)*	0 (0–300)	1500 (1000–2000)	1400 (1000–2000)	NA	NA	4470 (NA)	3916 (NA)
Ackland [47]	500 (0–1000)	625 (0–1438)	3000 (2000–4000)	3000 (2000–4000)	NA	NA	5000 (NA)	4748 (NA)
Correa-Gallego [48]	NA	NA	NA	NA	NA	NA	3800 (NA)	4500 (NA)
Weinberg [34]	200 (500–700)	200 (175–550)	1875 (1000–2000)	4000 (2313–4206)*	NA	NA	4516 (NA)	7034 (NA)
Gomez-Izquierdo [49]	500 (323–687)	2102 (1600–2528)*	900 (400–1400)*	0 (0–500)	NA	NA	1819 (NA)	2914 (NA)
Wu [50]	900 (400–1400)*	0 (0–500)	500 (323–687)	2102 (1600–2528)	NA	NA	1535 (1000–2272)	2370 (1779–3071)
Zhao [33]	775 (539–1011)*	487 (435–539)	471 (371–571)	459 (427–491)	885 (377)	443 (140)	1478 (1167–1790)	1183 (1120–1246)
Weinberg yyy[29]	200 (500–700)	200 (175–550)	1875 (1000–2000)	2000 (1125–2000)	356 (248–465)	465 (465–465)	4950 (NA)	4450 (NA)

NA, data not available; GDT, patients’ subgroup receiving goal-directed therapy; ml, milliliters. Data are reported including 95% confidence interval (95%CI) or standard deviation (SD), as appropriate; when NA is present after a value, it implies a computation of available data reported in the paper. Perioperative fluid therapy reports the overall amount of fluid includes the fluid infused during the operation and within the first 24 h in the critically ill area. * = difference reported in the study as statistically significant among GDT/controls subgroups regarding intraoperative colloids or crystalloids administration

Second, the overall amount of perioperative fluid administration may be biased by the lacking report of oral fluid intake or perioperative maintenance fluid (i.e., sometime reported as ml/kg/hr, without providing data regarding the median weight of the population and/or the hours of observation).

Third, the study is limited due to concerns regarding bias in most of the included studies. The different sample sizes of the included studies may add another source of heterogeneity (as confirmed by the interquartile ranges of the *I*^2^). Additionally, the overall quality of the included studies reported “some concerns” in the majority (76.2%), mostly related to the selection of the reported results due to drawbacks in the trial registration. Moreover, the definition of postoperative complications and the timing in mortality assessment may vary among the included studies, implying a bias in the comparability of the reported outcomes.

Finally, according to previous meta-analyses in this field [8, 40], we adopted a database combination search strategy including PUBMED®, EMBASE® and the Cochrane Controlled Clinical trials register, excluding different sources (*i.e.* Web of Science®). Although this choice should allow a reliable coverage of the published studies for the topic of interest, some RCTs could not be identified.

Despite the abovementioned limitations and the marked heterogeneity in trial quality and design, our results suggest that GDT in the perioperative settings has a significant benefit in reducing rates of complications. This study strengthens the concept that GDT should be routinely applied in perioperative settings, especially in those surgeries in need of high intravascular volume replacement.

## Conclusions

GDT strategy reduces postoperative complications, but not perioperative mortality, irrespectively of perioperative overall amount of fluids infused.

## Supplementary information

**Additional file 1**. **Table S1**: PRISMA-DTA checklist. **Table S2**: Extracted data in each study assessed for eligibility. **Table S3**: Full text articles excluded, not fitting eligibility criteria. **Table S4**: Summary of perioperative cumulative fluid balance in the included studies. **Table S5**: Renal complications. **Table S6**: Cardiovascular complications. **Table S7**: Pulmonary complications. **Table S8**: Neurological complications. **Table S9**: Infective complications. **Figure S1**: Risk of bias assessment of the included studies. **Figure S2**: Bias assessment plot. Overall complications. **Figure S3**: Bias assessment plot. Overall mortality.

## Data Availability

The datasets used and/or analyzed during the current study are available from the corresponding author on reasonable request.

## Abbreviations

GDT: goal-directed therapy
RCT: randomized-controlled trial
ERAS: enhanced recovery after surgery
SD: standard deviation
95% CI: 95% confidence intervals
