# Supplementary material for: Association between perioperative fluid administration and postoperative outcomes: a 20-year systematic review and a meta-analysis of randomized goal-directed trials in major visceral/noncardiac surgery
Source: Crit Care. 2021 Feb 1;25:43. doi: 10.1186/s13054-021-03464-1 (PMC7849093; doi:10.1186/s13054-021-03464-1)
Supplement: Supplementary file 1 — Additional file 1. Table S1: PRISMA-DTA checklist. Table S2: Extracted data in each study assessed for eligibility. Table S3: Full text articles excluded, not fitting eligibility criteria. Table S4: Summary of perioperative cumulative fluid balance in the included studies. Table S5: Renal complications. Table S6: Cardiovascular complications. Table S7: Pulmonary complications. Table S8: Neurological complications. Table S9: Infective complications. Figure S1: Risk of bias assessment of the included studies. Figure S2: Bias assessment plot. Overall complications. Figure S3: Bias assessment plot. Overall mortality. [file 13054_2021_3464_MOESM1_ESM.docx]

**Association between perioperative fluid administration and postoperative outcomes: a 20-years systematic review and a meta-analysis of randomized goal-directed trials in major visceral/non-cardiac surgery.**

Informative title: perioperative fluid strategies and postoperative outcomes

Antonio Messina, MD, PhD^1,2^; Chiara Robba, MD, PhD^3^; Lorenzo Calabrò, MD^1^; Daniel Zambelli, MD^1^; Francesca Iannuzzi, MD^3,4^; Edoardo Molinari, MD^3,4^; Silvia Scarano, MD^3,4^; Denise Battaglini, MD^3^; Marta Baggiani, MD^5^; Giacomo De Mattei^6^; Laura Saderi, BsC^7^; Giovanni Sotgiu MD, PhD^7^; Paolo Pelosi, MD, FERS^3,4^; Maurizio Cecconi MD, FRCA, FICM^1,2^.

^1^Humanitas Clinical and Research Center – IRCCS, Rozzano, MI, Italy; ^2^Department of Biomedical Sciences, Humanitas University, Pieve Emanuele, MI, Italy; ^3^Anaesthesia and Intensive Care, San Martino Policlinico Hospital, IRCCS for Oncology and Neuroscience, Genoa, Italy; ^4^Department of Surgical Sciences and Integrated Diagnostic (DISC), University of Genoa, Genoa, Italy; ^5^Anesthesia and Intensive Care Medicine, Maggiore della Carità University Hospital, Novara, Italy; ^6^Anesthesia and Intensive Care Medicine, Azienda Sanitaria Universitaria Integrata Udine, Italy; ^7^Clinical Epidemiology and Medical Statistics Unit, Departement of Medical, Surgical and Experimental, University of Sassari

**Corresponding author:**

Antonio Messina; Department of Anaesthesia and Intensive Care Medicine

Humanitas Clinical and Research Center – IRCCS

Via Alessandro Manzoni, 56

20089 – Rozzano (MI) - Italy

Email: [antonio.messina@humanitas.it](mailto:antonio.messina@humanitas.it)

**SUPPLEMENTAL MATERIALS**

**Table of Contents**

**Table S1** PRISMA-DTA checklist.

**Table S2** Extracted data in each study assessed for eligibility.

**Table S3** Full-text articles excluded, not fitting eligibility criteria.

**Table S4.** Summary of perioperative cumulative fluid balance in the included studies.

**Table S5.** Renal complications.

**Table S6.** Cardiovascular complications.

**Table S7.** Pulmonary complications.

**Table S8.** Neurological complications.

**Table S9.** Infective complications.

**Figure S1** Risk of bias assessment of the included studies

**Figure S2.** Bias assessment plot. Overall complications.

**Figure S3.** Bias assessment plot. Overall mortality.

**Table S1. PRISMA-DTA checklist for systematic review and meta-analysis**

| **Section/topic** | **#** | **Checklist item** | **Reported on page #** |
| --- | --- | --- | --- |
| **TITLE** | | |  |
| Title | 1 | Identify the report as a systematic review, meta-analysis, or both. | 1 |
| **ABSTRACT** | | |  |
| Structured summary | 2 | Provide a structured summary including, as applicable: background; objectives; data sources; study eligibility criteria, participants, and interventions; study appraisal and synthesis methods; results; limitations; conclusions and implications of key findings; systematic review registration number. | 2-3 |
| **INTRODUCTION** | | |  |
| Rationale | 3 | Describe the rationale for the review in the context of what is already known. | 4-5 |
| Objectives | 4 | Provide an explicit statement of questions being addressed with reference to participants, interventions, comparisons, outcomes, and study design (PICOS). | 4-5 |
| **METHODS** | | |  |
| Protocol and registration | 5 | Indicate if a review protocol exists, if and where it can be accessed (e.g., Web address), and, if available, provide registration information including registration number. | 6 |
| Eligibility criteria | 6 | Specify study characteristics (e.g., PICOS, length of follow-up) and report characteristics (e.g., years considered, language, publication status) used as criteria for eligibility, giving rationale. | 6 |
| Information sources | 7 | Describe all information sources (e.g., databases with dates of coverage, contact with study authors to identify additional studies) in the search and date last searched. | 6-7 |
| Search | 8 | Present full electronic search strategy for at least one database, including any limits used, such that it could be repeated. | 6-7 |
| Study selection | 9 | State the process for selecting studies (i.e., screening, eligibility, included in systematic review, and, if applicable, included in the meta-analysis). | 7 |
| Data collection process | 10 | Describe method of data extraction from reports (e.g., piloted forms, independently, in duplicate) and any processes for obtaining and confirming data from investigators. | 7 |
| Data items | 11 | List and define all variables for which data were sought (e.g., PICOS, funding sources) and any assumptions and simplifications made. | 8-9 |
| Risk of bias in individual studies | 12 | Describe methods used for assessing risk of bias of individual studies (including specification of whether this was done at the study or outcome level), and how this information is to be used in any data synthesis. | 8 |
| Summary measures | 13 | State the principal summary measures (e.g., risk ratio, difference in means). | 8-9 |
| Synthesis of results | 14 | Describe the methods of handling data and combining results of studies, if done, including measures of consistency (e.g., I^2^) for each meta-analysis. | 8-9 |

Page 1 of 2

| **Section/topic** | **#** | **Checklist item** | **Reported on page #** |
| --- | --- | --- | --- |
| Risk of bias across studies | 15 | Specify any assessment of risk of bias that may affect the cumulative evidence (e.g., publication bias, selective reporting within studies). | 10 |
| Additional analyses | 16 | Describe methods of additional analyses (e.g., sensitivity or subgroup analyses, meta-regression), if done, indicating which were pre-specified. | 10-11 |
| **RESULTS** | | |  |
| Study selection | 17 | Give numbers of studies screened, assessed for eligibility, and included in the review, with reasons for exclusions at each stage, ideally with a flow diagram. | 10 |
| Study characteristics | 18 | For each study, present characteristics for which data were extracted (e.g., study size, PICOS, follow-up period) and provide the citations. | 10 |
| Risk of bias within studies | 19 | Present data on risk of bias of each study and, if available, any outcome level assessment (see item 12). | Figure S1 |
| Results of individual studies | 20 | For all outcomes considered (benefits or harms), present, for each study: (a) simple summary data for each intervention group (b) effect estimates and confidence intervals, ideally with a forest plot. | 11-12 |
| Synthesis of results | 21 | Present results of each meta-analysis done, including confidence intervals and measures of consistency. | 11-12 |
| Risk of bias across studies | 22 | Present results of any assessment of risk of bias across studies (see Item 15). | Figure S1 |
| Additional analysis | 23 | Give results of additional analyses, if done (e.g., sensitivity or subgroup analyses, meta-regression [see Item 16]). | 11-12 |
| **DISCUSSION** | | |  |
| Summary of evidence | 24 | Summarize the main findings including the strength of evidence for each main outcome; consider their relevance to key groups (e.g., healthcare providers, users, and policy makers). | 13-14 |
| Limitations | 25 | Discuss limitations at study and outcome level (e.g., risk of bias), and at review-level (e.g., incomplete retrieval of identified research, reporting bias). | 15-16-17 |
| Conclusions | 26 | Provide a general interpretation of the results in the context of other evidence, and implications for future research. | 18 |
| **FUNDING** | | |  |
| Funding | 27 | Describe sources of funding for the systematic review and other support (e.g., supply of data); role of funders for the systematic review. | 20 |

**Table S2. Extracted data in each study assessed for eligibility.**

| Study Reference | Names and surnames of authors, year of publication. |
| --- | --- |
| Country | Country/countries in which the study was carried out. |
| Study design and type of surgery | Modality of recruitment  Surgical setting where the study has been performed |
| Gender | Percentage of male patients |
| Age | Patient age reported in the study (as mean ± sd or median). |
| Patients’ characteristics and peri-operatory risk. | Body mass index; height; weight (as mean ± sd or median). American Society of Anaesthesiologists physical status. |
| Patients’ enrollment | Number of enrolled/eligible/dropped out patients, in the two study groups |
| Peri-operatory fluids | Cumulative Intraoperative, postoperative, overall fluid volume infusions (colloids or crystalloids), as mean ± sd or median). |
| Mortality | Mortality rate reported with definition. |
| Complications | Expressed as overall number of events reported as percentage of patients who had at least one complication |

**Table S3. Full text articles excluded, not fitting eligibility criteria.**

| **Excluded Studies** | **Reason for exclusion** |
| --- | --- |
| Senagore et al. 2009 | Colloid versus crystalloid comparison |
| Bonazzi et al. 2002 | GDT only pre-operative |
| Harten et al 2008 | Not elective surgery |
| Cecconi et al. 2011 | Orthopedic surgery |
| Van der Linden et al 2010 | Complications not reported |
| Jhanji et al. 2010 | Complications not reported |
| Venn et al. 2002 | Orthopedic surgery |
| Wu et al. 2017 | GDT comparison (two types of GDT compared) |
| Coeckelenbergh et al 2019 | GDT comparison (two types of GDT compared) |
| Zhang et al. 2012 | Colloid versus crystalloid comparison |
| Buettner et al. 2008 | Complications rate not reported |
| Pillai et al. 2011 | Fluid volumes not reported |
| Melis et al. 2012 | Retrospective |
| Wang et al. 2014 | No RCT |
| Kuleman et al. 2017 | No RCT |
| McArdle et al. 2009 | Unblinded |
| Barak et al. 2006 | Complications rate not reported |
| Yates et al. 2014 | Colloid versus crystalloid comparison |
| Kumar et al. 2016 | GDT comparison (two types of GDT compared) |
| Healy et al. 2016 | No RCT |
| Sandham et al 2003 | Complications rate not reported |
| Mackay et al. 2016 | Complications rate not reported |
| Holte et al. 2004 | Complications rate not reported |
| Gan et al. 2002 | Complication rate not reported |
| Noblett et al. 2006 | Complication rate not reported |
| Forget et al. 2010 | Complication rate not reported |
| Challand et al. 2012 | Complication rate not reported |
| Zheng et al. 2013 | Complication rate not reported |
| Colantonio et al. 2015 | Complication rate not reported |
| Harten et al 2008 | Non elective surgery |
| Xu et al. 2017 | Complication rate not reported |
| Gerent et al. 2018 | Complication rate not reported |
| Yin et al. 2018 | Complication rate not reported |
| Luo et al. 2017 | Neurosurgery |
|  |  |

GDT, goal-directed therapy; RCT, randomized controlled study. In the studies excluded for “complication rate not reported”, there was not a clear statement of the overall rate of complication, whereas the overall number of organ-specific complications may be stated.

**Table S4. Summary of perioperative cumulative fluid balance in the included studies.**

| **Study** | **Cumulative**  **Fluid balance D0 (mL)** | | **Cumulative**  **Fluid balance D1 (mL)** | |
| --- | --- | --- | --- | --- |
|  |  |  |  |  |
|  | **GDT** | **Controls** | **GDT** | **Controls** |
| Conway D.H.^1^ | NA | NA | NA | NA |
| Wakeling H.G.^2^ | NA | NA | NA | NA |
| Pearse R.^3^ | NA | NA | NA | NA |
| Lobo S.M.^4^ | NA | NA | NA | NA |
| Donati A.^5^ | NA | NA | NA | NA |
| Benes J.^6^ | NA | NA | NA | NA |
| Mayer J.^7^ | NA | NA | NA | NA |
| Brandstrup B.^8^ | NA | NA | NA | NA |
| Salzwedel C.^9^ | 3956 (2469) | 4332 (3715) | NA | NA |
| McKenny M.^10^ | NA | NA | NA | NA |
| Scheeren T.W.^11^ | NA | NA | NA | NA |
| Srinivasa S.^12^ | NA | NA | NA | NA |
| Pearse RM.^13^ | NA | NA | NA | NA |
| Phan T.D.^14^ | NA | NA | NA | NA |
| Ackland G.L.^15^ | NA | NA | NA | NA |
| Correa-Gallego C.^16^ | NA | NA | NA | NA |
| Weinberg L.^17^ | 1005 (475-1873**)** mL | 3300 (2474-3874) mL | 1661 (1253-2041) mL | 1177 (704-1725) mL |
| Gomez-Izquierdo J.C.^18^ | NA | NA | NA | NA |
| Wu J.^19^ | NA | NA | NA | NA |
| Zhao G.^20^ | NA | NA | NA | NA |
| Weinberg L.^21^ | 808 (571-1565) | 1345 (900-1983) | 1535 (757-2238) mL | 1727 (1072-2350) mL |

NA, data not available; GDT, goal-directed therapy; D0, day of the operation; D1, first postoperative day. Data are reported, including 95% confidence interval (95%CI) or standard deviation (SD), as appropriate.

**Table S5. Renal complications.**

**Pooled risk difference and heterogeneity (I^2^) assessment.**

| **Study** | **Risk difference (95% CI)** |
| --- | --- |
| Wakeling H.G. et al, 2005 | 0.0253 (-0.057;0.1251) |
| Lobo S.M. et al, 2006 | -0.0385 (-0.1734;0.1222) |
| Donati A. et al, 2007 | -0.037 (-0.3021;0.3271) |
| Jan B. et al, 2010 | 0.0253 (-0.0792;0.181) |
| Mayer J. et al, 2010 | -0.0432 (-0.1755;0.1787) |
| Brandstrup B. et al, 2012 | 0 (-0.093;0.1001) |
| McKenny M. et al, 2013 | 0 (-0.2112;0.3342) |
| Srinivasa S, et al, 2013 | 0.0043 (-0.1877;0.1999) |
| Pearse RM. et al, 2014 | 0.0069 (-0.0354;0.051) |
| Phan T.D. et al, 2014 | 0 (-0.3217;0.8102) |
| Correa-Gallego C. et al, 2015 | 0.0283 (-0.0424;0.1075) |
| Weinberg L. et al, 2017 | 0.0366 (-0.0503;0.1628) |
| **Pooled risk difference** | **0.0082 (-0.0169; 0.0333)** |
| I_2_ (95% CI) | 0% (0%-46.4%) |
| Chi_2_ (test risk difference differs from 0) = 0.4078 (df = 1) p-value **=** 0.52 | |

95%CI, 95% Confidence interval.

**Table S6. Cardiovascular complications.**

**Pooled risk difference and heterogeneity (I^2^) assessment.**

| **Study** | **Risk difference (95% CI)** |
| --- | --- |
| Wakeling H.G. et al, 2005 | 0.0118 (-0.1156;0.1478) |
| Pearse R. et al, 2005 | 0.0083 (-0.1221;0.1648) |
| Lobo S.M. et al, 2006 | -0.1795 (-0.3723;0.0437) |
| Donati A. et al, 2007 | 0.2593 (-0.062;0.5846) |
| Jan B. et al, 2010 | 0.0231 (-0.1218;0.205) |
| Mayer J. et al, 2010 | -0.0912 (-0.2999;0.1777) |
| Brandstrup B. et al, 2012 | 0.0902 (-0.0534;0.2509) |
| McKenny M. et al, 2013 | -0.0083 (-0.2961;0.3715) |
| Pearse RM. et al, 2014 | 0.0225 (-0.0501;0.0957) |
| Phan T.D. et al, 2014 | -0.3333 (-0.6596;0.555) |
| Weinberg L. et al, 2017 | -0.0198 (-0.0996;0.0921) |
| Wu J. et al, 2017 | -0.2727 (-0.5751;0.2837) |
| Zhao G. et al, 2018 | -0.0438 (-0.1597;0.0807) |
| Weinberg L. et al, 2019 | 0.0417 (-0.2208;0.2992) |
| **Pooled risk difference** | **0.0441 (0.0051; 0.0831)** |
| I_2_ (95% CI) | 0% (0%-47.4%) |
| Chi_2_ (test risk difference differs from 0) = 0.0331 (df = 1) p-value = 0.86 | |

95%CI, 95% Confidence interval.

**Table S7. Pulmonary (non-infective) complications.**

**Pooled risk difference and heterogeneity (I^2^) assessment.**

| **Study** | **Risk difference (95% CI)** |
| --- | --- |
| Wakeling H.G. et al, 2005 | 0.1027 (-0.0016;0.2273) |
| Pearse R. et al, 2005 | -0.0191 (-0.1115;0.1057) |
| Lobo S.M. et al, 2006 | 0 (-0.0909;0.1305) |
| Donati A. et al, 2007 | -0.037 (-0.2511;0.3107) |
| Jan B. et al, 2010 | 0.0193 (-0.1149;0.1944) |
| Mayer J. et al, 2010 | 0.0564 (-0.08;0.2904) |
| Brandstrup B. et al, 2012 | 0 (-0.093;0.1001) |
| McKenny M. et al, 2013 | -0.0083 (-0.2961;0.3715) |
| Pearse RM. et al, 2014 | 0.0099 (-0.017;0.0403) |
| Phan T.D. et al, 2014 | 0 (-0.3217;0.8102) |
| Weinberg L. et al, 2017 | 0.0267 (-0.0738;0.1614) |
| Wu J. et al, 2017 | 0.3182 (-0.1724;0.7359) |
| Zhao G. et al, 2018 | 0.0244 (-0.0436;0.1268) |
| Weinberg L. et al, 2019 | -0.0417 (-0.2596;0.1756) |
| **Pooled risk difference** | **0.0189 (-0.0062; 0.0441)*** |
| I_2_ (95% CI) | 0% (0%-47.4%) |
| Chi_2_ (test risk difference differs from 0) = 2.1708 (df = 1) p-value = 0.14 | |

95%CI, 95% Confidence interval.

**Table S8. Neurological complications.**

**Pooled risk difference and heterogeneity (I^2^) assessment.**

| **Study** | **Risk difference (95% CI)** |
| --- | --- |
| Wakeling H.G. et al, 2005 | -0.0236 (-0.115;0.0724) |
| Pearse R. et al, 2005 | 0.0354 (-0.0218;0.145) |
| Lobo S.M. et al, 2006 | 0 (-0.0909;0.1305) |
| Jan B. et al, 2010 | -0.0137 (-0.0741;0.0949) |
| Mayer J. et al, 2010 | -0.0204 (-0.108;0.1673) |
| Brandstrup B. et al, 2012 | 0 (-0.093;0.1001) |
| McKenny M. et al, 2013 | 0 (-0.2112;0.3342) |
| Pearse RM. et al, 2014 | -0.0126 (-0.0413;0.0149) |
| Phan T.D. et al, 2014 | -0.1111 (-0.454;0.73) |
| Weinberg L. et al, 2017 | 0.0237 (-0.0393;0.132) |
| Wu J. et al, 2017 | 0 (-0.2723;0.5071) |
| Zhao G. et al, 2018 | 0 (-0.0671;0.0865) |
| Weinberg L. et al, 2019 | -0.0833 (-0.2785;0.0984) |
| **Pooled risk difference** | **-0.0069 (0.0245; 0.0107)** |
| I_2_ (95% CI) | 0% (0%-51.2%) |
| Chi_2_ (test risk difference differs from 0) = 0.5855 (df = 1) p-value = 0.44 | |

95%CI, 95% Confidence interval.

**Table S9. Infective complications.**

**Pooled risk difference and heterogeneity (I^2^) assessment.**

| **Study** | **Risk difference (95% CI)** |
| --- | --- |
| Wakeling H.G. et al, 2005 | 0.0926 (-0.0541;0.2446) |
| Pearse R. et al, 2005 | -0.0884 (-0.2648;0.0896) |
| Lobo S.M. et al, 2006 | 0.2564 (0.0072;0.4707) |
| Jan B. et al, 2010 | -0.265 (-0.4514;-0.0585) |
| Mayer J. et al, 2010 | -0.012 (-0.2342;0.2569) |
| Brandstrup B. et al, 2012 | -0.091 (-0.3099;0.1362) |
| Salzwedel C. et al, 2013 | -0.1111 (-0.2669;0.0567) |
| McKenny M. et al, 2013 | -0.2833 (-0.603;0.1506) |
| Srinivasa S, et al, 2013 | -0.067 (-0.2934;0.1658) |
| Pearse RM. et al, 2014 | -0.1774 (-0.2585;-0.0936) |
| Phan T.D. et al, 2014 | -0.1111 (-0.454;0.73) |
| Correa-Gallego C. et al, 2015 | 0.0626 (-0.0869;0.2093) |
| Weinberg L. et al, 2017 | 0.1097 (-0.032;0.271) |
| Wu J. et al, 2017 | -0.1818 (-0.4876;0.3603) |
| Zhao G. et al, 2018 | -0.1594 (-0.3501;0.0444) |
| Weinberg L. et al, 2019 | -0.125 (-0.3611;0.1199) |
| **Pooled risk difference** | **-0.0591 (-0.1326; 0.0143)** |
| I_2_ (95% CI) | 55.8% (9.1% to 73.5%) |
| Chi_2_ (test risk difference differs from 0) = 2.4888 (df = 1) p-value = 0.12 | |

95%CI, 95% Confidence interval.

**Figure S1. Risk of bias assessment of the included studies**

**
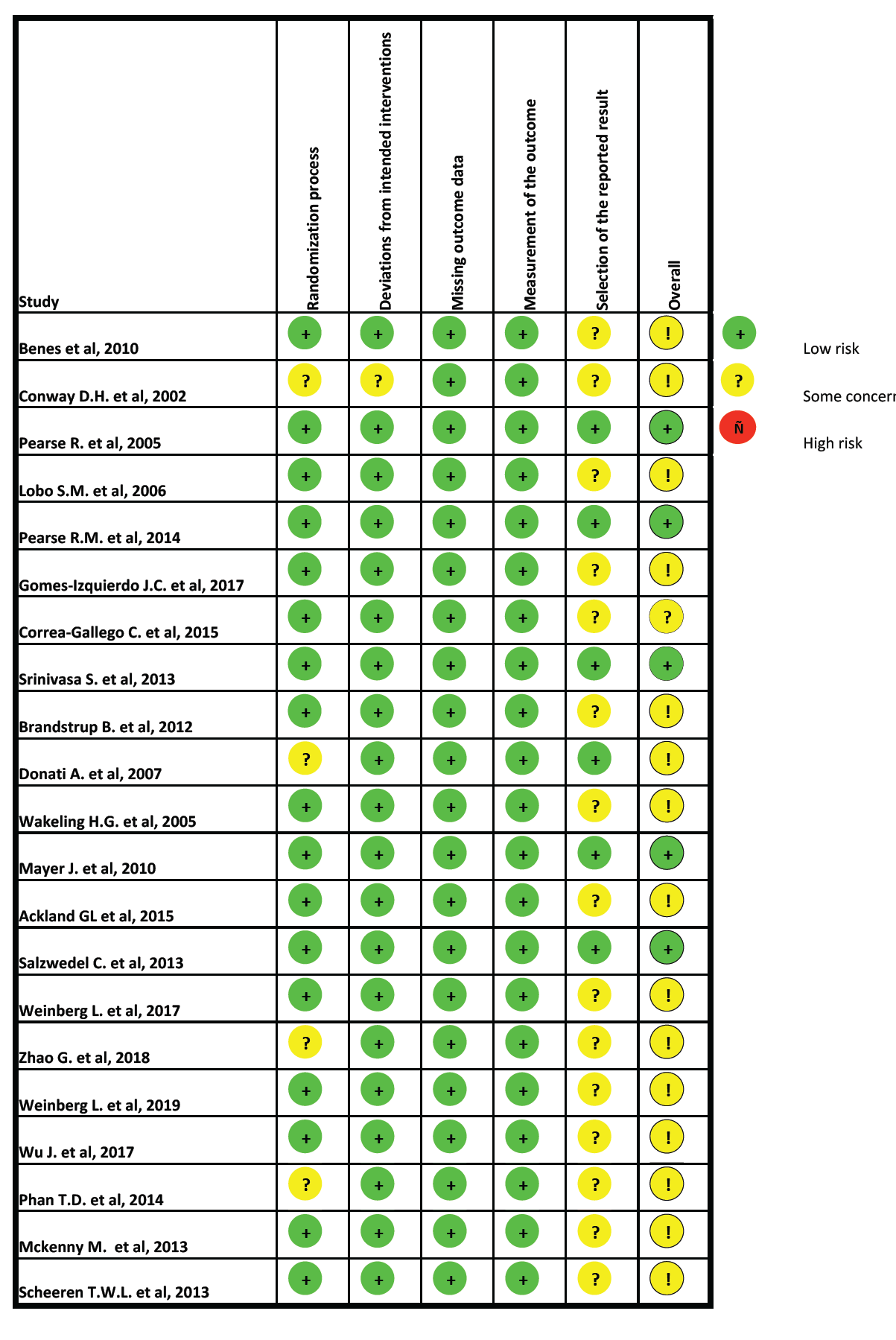
**

**Figure S2. Bias assessment plot.**

**Overall complications.**

**Figure S3. Bias assessment plot.**

**Overall mortality**

**Bibliography of Supplemental Materials**

1 . Conway DH, Mayall R, Abdul-Latif MS, Gilligan S, Tackaberry C. Randomised controlled trial investigating the influence of intravenous fluid titration using oesophageal Doppler monitoring during bowel surgery. *Anaesthesia* 2002; **57**: 845-9

2 . Wakeling HG, McFall MR, Jenkins CS, et al. Intraoperative oesophageal Doppler guided fluid management shortens postoperative hospital stay after major bowel surgery. *Br J Anaesth* 2005; **95**: 634-42

3 . Pearse R, Dawson D, Fawcett J, Rhodes A, Grounds RM, Bennett ED. Early goal-directed therapy after major surgery reduces complications and duration of hospital stay. A randomised, controlled trial [ISRCTN38797445]. *Crit Care* 2005; **9**: R687-93

4 . Lobo SM, Lobo FR, Polachini CA, et al. Prospective, randomized trial comparing fluids and dobutamine optimization of oxygen delivery in high-risk surgical patients [ISRCTN42445141]. *Crit Care* 2006; **10**: R72

5 . Donati A, Loggi S, Preiser JC, et al. Goal-directed intraoperative therapy reduces morbidity and length of hospital stay in high-risk surgical patients. *Chest* 2007; **132**: 1817-24

6 . Benes J, Chytra I, Altmann P, et al. Intraoperative fluid optimization using stroke volume variation in high risk surgical patients: results of prospective randomized study. *Crit Care* 2010; **14**: R118

7 . Mayer J, Boldt J, Mengistu AM, Rohm KD, Suttner S. Goal-directed intraoperative therapy based on autocalibrated arterial pressure waveform analysis reduces hospital stay in high-risk surgical patients: a randomized, controlled trial. *Crit Care* 2010; **14**: R18

8 . Brandstrup B, Svendsen PE, Rasmussen M, et al. Which goal for fluid therapy during colorectal surgery is followed by the best outcome: near-maximal stroke volume or zero fluid balance? *Br J Anaesth* 2012; **109**: 191-9

9 . Salzwedel C, Puig J, Carstens A, et al. Perioperative goal-directed hemodynamic therapy based on radial arterial pulse pressure variation and continuous cardiac index trending reduces postoperative complications after major abdominal surgery: a multi-center, prospective, randomized study. *Crit Care* 2013; **17**: R191

10 . McKenny M, Conroy P, Wong A, et al. A randomised prospective trial of intra-operative oesophageal Doppler-guided fluid administration in major gynaecological surgery. *Anaesthesia* 2013; **68**: 1224-31

11 . Scheeren TW, Wiesenack C, Gerlach H, Marx G. Goal-directed intraoperative fluid therapy guided by stroke volume and its variation in high-risk surgical patients: a prospective randomized multicentre study. *J Clin Monit Comput* 2013; **27**: 225-33

12 . Srinivasa S, Taylor MH, Singh PP, Yu TC, Soop M, Hill AG. Randomized clinical trial of goal-directed fluid therapy within an enhanced recovery protocol for elective colectomy. *Br J Surg* 2013; **100**: 66-74

13 . Pearse RM, Harrison DA, MacDonald N, et al. Effect of a perioperative, cardiac output-guided hemodynamic therapy algorithm on outcomes following major gastrointestinal surgery: a randomized clinical trial and systematic review. *JAMA* 2014; **311**: 2181-90

14 . Phan TD, D'Souza B, Rattray MJ, Johnston MJ, Cowie BS. A randomised controlled trial of fluid restriction compared to oesophageal Doppler-guided goal-directed fluid therapy in elective major colorectal surgery within an Enhanced Recovery After Surgery program. *Anaesth Intensive Care* 2014; **42**: 752-60

15 . Ackland GL, Iqbal S, Paredes LG, et al. Individualised oxygen delivery targeted haemodynamic therapy in high-risk surgical patients: a multicentre, randomised, double-blind, controlled, mechanistic trial. *Lancet Respir Med* 2015; **3**: 33-41

16 . Correa-Gallego C, Tan KS, Arslan-Carlon V, et al. Goal-Directed Fluid Therapy Using Stroke Volume Variation for Resuscitation after Low Central Venous Pressure-Assisted Liver Resection: A Randomized Clinical Trial. *J Am Coll Surg* 2015; **221**: 591-601

17 . Weinberg L, Ianno D, Churilov L, et al. Restrictive intraoperative fluid optimisation algorithm improves outcomes in patients undergoing pancreaticoduodenectomy: A prospective multicentre randomized controlled trial. *PLoS One* 2017; **12**: e0183313

18 . Gómez-Izquierdo JC, Trainito A, Mirzakandov D, et al. Goal-directed Fluid Therapy Does Not Reduce Primary Postoperative Ileus after Elective Laparoscopic Colorectal Surgery: A Randomized Controlled Trial. *Anesthesiology* 2017; **127**: 36-49

19 . Jie Wu YM, Tianlong Wang, Geng Xu, Long Fan, Ying Zhang. Goal-directed fluid management based on the auto-calibrated arterial pressure-derived stroke volume variation in patients undergoing supratentorial neoplasms surgery. *Int J Clin Exp Med* 2017; **10**: 3106-14

20 . Guoliang Zhao* PP, Yinyan Zhou, Junjie Li, Haiyan Jiang, Jianlin Shao. The accuracy and effectiveness of goal directed

fluid therapy in plateau-elderly gastrointestinal cancer patients: a prospective randomized controlled trial. *Int J Clin Exp Med* 2018; **11**: 8516-22

21 . Weinberg L, Ianno D, Churilov L, et al. Goal directed fluid therapy for major liver resection: A multicentre randomized controlled trial. *Ann Med Surg (Lond)* 2019; **45**: 45-53

22 . Luo J, Xue J, Liu J, Liu B, Liu L, Chen G. Goal-directed fluid restriction during brain surgery: a prospective randomized controlled trial. *Ann Intensive Care* 2017; **7**: 16
